# *Chlamydia trachomatis* Growth and Cytokine mRNA Response in a Prostate Cancer Cell Line

**DOI:** 10.1155/2019/6287057

**Published:** 2019-01-17

**Authors:** Ivan M. Petyaev, Naylia A. Zigangirova, Elena Y. Morgunova, Nigel H. Kyle, Elena D. Fedina, Yuriy K. Bashmakov

**Affiliations:** ^1^Lycotec Ltd., Granta Park, Cambridge CB21 6GP, UK; ^2^Department of Medical Microbiology, Gamaleya National Research Center of Epidemiology and Microbiology, Ministry of Health, 18 Gamaleya Str, Moscow 123098, Russia

## Abstract

In the present paper, we report that *C. trachomatis* can be efficiently propagated and affect mRNA expression for two major cytokines, relevant to tumor progression, in CWR-R1 cells, a malignant prostate cell line. CWR-R1 and McCoy cells, a classic cell line for chlamydial research, were grown and infected with *C. trachomatis* under similar conditions. Cell monolayers were harvested for RNA analysis and immunostaining with major outer membrane protein (MOMP) antibody at 24, 48, and 72 hours of the postinfection (hpi) period. It was shown that the infectious cycle of chlamydial pathogen in CWR-R1 cells resembles the progression of *C. trachomatis* infection in McCoy cells but with a few important differences. First of all, the initial stage of *C. trachomatis* propagation in CWR-R1 cells (24 hpi) was characterized by larger inclusion bodies and more intense, specific immunofluorescent staining of infected cells as compared with McCoy cells. Moreover, there was a corresponding increase in infective progeny formation in CWR-R1 cells along with mRNA for EUO, a crucial gene controlling the early phase of the chlamydial development cycle (24 hpi). These changes were more minimal and became statistically insignificant at a later time point in the infectious cycle (48 hpi). Altogether, these data suggest that the early phase of *C. trachomatis* infection in CWR-R1 cells is accompanied by more efficient propagation of the pathogen as compared with the growth of *C. trachomatis* in McCoy cells. Furthermore, propagation of *C. trachomatis* in CWR-R1 cells leads to enhanced transcription of interleukin-6 and fibroblast growth factor-2, genes encoding two important proinflammatory cytokines implicated in the molecular mechanisms of chemoresistance of prostate cancer and its ability to metastasize. The possible roles of reactive oxygen species and impaired mitochondrial oxidation in the prostate cancer cell line are discussed as factors promoting the early stages of *C. trachomatis* growth in CWR-R1 cells.

## 1. Introduction

*Chlamydia trachomatis* (*C. trachomatis*) is an obligate, nonspore-forming intracellular Gram-negative bacterial pathogen able to propagate in epitheliocytes of mucous membranes (urogenital system and eye) and which displays a distinct parasitic cell cycle [1, 2]. *C. trachomatis* is the most prevalent sexually transmitted pathogen causing a wide range of syndromes and diseases, with an alarming growth rate of 100 million newly reported cases annually worldwide [3, 4]. Although most *C. trachomatis* infections are asymptomatic, untreated individuals may develop a variety of genital (urethritis, endocervicitis, salpingitis, endometritis, and pelvic inflammatory disease) and extragenital (arthritis, perihepatitis, and ocular infection) manifestations [5]. There are 17 *C. trachomatis* serovars. All of them have a similar and unique life cycle and exist in two developmental forms—elementary body and reticulate body [6]. Eukaryotic cells are infected with nondividing elementary bodies which form phagolysosomal fusion particles in the cytoplasm of the host cell at the initial stage of infection. Inside, the endosome elementary bodies differentiate into reticulate bodies which divide via binary fission [1, 7]. Once infection progresses and the endosome (otherwise known as the inclusion body) accumulates a significant number of reticulate bodies, a reverse transformation of reticulate bodies into elementary bodies takes place. Newly formed elementary bodies undergo exocytosis after 48–72 hours of postinfection development to initiate a new round of *C. trachomatis* infection in neighboring cells. Such a sophisticated developmental cycle is highly energy dependent and known to be supported by host cell metabolism [8]. All members of Chlamydiaceae rely heavily on the host cell metabolism. The chlamydial genome lacks most of the genes encoding cholesterol biosynthesis although chlamydial species can synthetize some phospholipids, long chain fatty acids and branched fatty acids [8–10]. Chlamydiaceae are completely auxotrophic for purine and pyrimidine nucleotides, most amino acids, and utilize host cell cytosolic ATP for at least the early stages of infection [11].

A large number of nonhematopoietic cells can support *C. trachomatis* growth using in vitro systems. Most of the genital strains grow very efficiently in a McCoy mouse fibroblast cell line, which is a classic cell for stock expansion and maintenance [12]. Ocular strains of the pathogen show preferential growth in conjunctival cells [13]. Epithelial cell lines from the vagina, endocervix, and endometrium have been established to provide physiologically relevant models for the study of pathogenesis in genital forms of *C. trachomatis* infection [14]. There is a continuous pursuit of physiologically relevant in vitro systems for the investigation of pathogenesis and treatment of *C. trachomatis* infection.

In the present paper, we report that *C. trachomatis* can be efficiently propagated and affect host cell mRNA expression for two major cytokines, relevant for tumor progression, in CWR-R1 cells, a malignant prostate cell line.

## 2. Materials and Methods

### 2.1. Reagents and Organisms

All reagents were from Sigma-Aldrich (St. Louis, USA) unless specified otherwise. *C. trachomatis* strain L2/Bu434 was kindly provided by Dr. P Saikku (University of Oulu, Finland). CWR-R1 cells (a human prostate cancer epithelial cell line), as well as McCoy cells (a mouse fibroblast cell line), were obtained from the Cultured Cells Depository Collection (Moscow, Russia).

### 2.2. Cell Culture

*C. trachomatis* was propagated in McCoy mouse fibroblasts grown in DMEM with 10% HyClone FCS supplemented with 2 mM glutamine, 4.0 mg/ml gentamicin, and 5.0 mg/ml amphotericin B, and further purified by Renografin gradient centrifugation, as previously described [15]. Briefly, elementary bodies were isolated and resuspended in sucrose-phosphate-glutamic acid (SPG) buffer (0.2 M sucrose, 8.6 mM Na2HPO4, 3.8 mM KH2PO4, 5 mM glutamic acid, 0.2 mm-filtered, pH 7.4). Titers were determined by infecting cell monolayers with dilutions of thawed stock suspension (2.5 × 10^7^). Subconfluent McCoy cell monolayers were infected with the suspension of *C. trachomatis* at MOI 1. Plates were centrifuged for 1 hour at 1500 g to synchronize the infection. Nucleic acid-based assay (RT-PCR) was performed at 24, 48, and 72 hours following *C. trachomatis* inoculation of the cultured cells. The infection rate in the McCoy cells and infective progeny formation was estimated as hours of postinfection (hpi) period following pathogen introduction into the incubation medium. To avoid discrepancies, CWR-R1 cells were grown, infected, and monitored under similar conditions.

### 2.3. Immunofluoresence Staining

Infected McCoy and CWR-R1 cells were grown on coverslips in 24-well plates and used for analysis at 0, 24, 48, and 72 hpi. The cells were fixed with ice-cold methanol, permeabilized with 0.1% Triton X-100, and preblocked for 1 hour at 4°C with 1% bovine serum albumin in PBS. Cell monolayers were stained using FITC-conjugated monoclonal antibody against MOMP, a major outer membrane protein (Bio-Rad, USA). Inclusion-containing cells were visualized using a Nikon Eclipse 50i microscope (Nikon, Japan) at ×1000 or lower magnification. Size of the inclusion bodies was measured using a MicroScan attachment to the microscope with a resolution of 0.1 *μ*m (Protvino, Russia) along with morphometric software (DiagNodus, Cambridge, UK).

### 2.4. Assessment of Infective Progeny

Infective progeny accumulation was assessed in McCoy and CWR-R1 cell monolayers infected with *C. trachomatis* at different time points. Infected cell monolayers were harvested 48 hours after bacterial inoculation and lysed by freezing/thawing. Serial dilutions of lysates were inoculated into McCoy cells, and plates were centrifuged for 1 hour at 1500 g. The infected cells were visualized at 48 hours of the postinfection period with FITC-conjugated monoclonal antibody against chlamydial MOMP, a major outer membrane protein (BioRad, USA).

#### 2.4.1. Attachment and Internalization Assay

This assay was performed as described in our previous paper [16].

### 2.5. RNA Extraction and Reverse Transcription

RNA was isolated from *C. trachomatis*-infected McCoy cells or CWR-R1 monolayers grown on 6-well plates using TRIZol (Invitrogen, Waltham MS, USA) at the 24-hour time point of the postinfection period. Total mRNA was pretreated with DNase I (DNA-free™, Ambion, Waltham MS, USA) and quantified on a NanoDrop ND-100 spectrophotometer (Thermo Fisher Scientific, Waltham MS, USA). 1 *μ*g of each RNA sample was converted into cDNA using random hexamer primers and a SuperScript III First-Strand Synthesis kit (Invitrogen, Waltham MS, USA).

#### 2.5.1. Quantitative Real-Time PCR

mRNA levels for *EUO*, a developmental gene of *C. trachomatis*, were analyzed in McCoy and CWR-R1 cells at different hpi by quantitative RT-PCR using a CFX-96 thermocycler and master mixes from Bio-Rad Laboratories (Hercules CA, USA). RT-qPCR Taqman primers were designed using Primer 3 software and were validated by BLAST search and regression plot analysis using Cp values obtained with multiple dilutions of cDNA. Specificity of the designed primers and fluorescent probes was confirmed in different systems using cDNA from *C. trachomatis* Bu434, *C. trachomatis* UW-3/Cx, *C. trachomatis* UW- 31/Cx, and *C. pneumoniae* K-6, as well as from uninfected McCoy and CWR-R1 cells and some other common bacterial pathogens as previously described [15].

The *C. trachomatis*-specific primers used were as follows: for *EUO* gene: Pr-F 5′ TCCCCGACGCTCTCCTTTCA 3′, Pr-R 5′CTCGTCAGGCTATCTATGTTGCT 3′, Probe 5′-ROX- ATG GAC GCC ACT TGT CCC ACG GAA T-BHQ2-3′. Primers for human interleukin-6 (IL-6) were as follows: PR-F 5′-CCAGCTATGAACTCCTTCTC-3′, PR-R 5′-GCTTGTTCCTCACATCTCTC-3′. Primers for human fibroblast growth factor-2 (FGF-2) were: PR-F 5′-GGCTTCTTCCTGCGCATCCA-3′ and PR-R 5′-GCTCTTAGCAGACATTGGAAGA-3′. Primer sequences for eukaryotic beta-actin were used as published previously [15, 16]. All primers were verified and used at 0.4 *µ*M final concentration under thermal cycling conditions of 95°C for 10 min, 50 cycles of 95°C for 15 seconds, 60°C for 1 min, and 72°C for 20 seconds. Serial dilutions of RNA extracted from C. *trachomatis*-infected McCoy and CWR-R1 cells were used as a standard for quantification of chlamydial gene expression. The mRNA expression levels were referenced to Ct values for chlamydial genes detected in uninfected McCoy cells grown under similar conditions. This reference value was taken as 1.00. All mRNA measurements were done in triplicate. All experiments were conducted at least three times. Statistical analysis was performed where possible using Student's *t*-test and calculation of medians as well as 95% confidence intervals. The most representative sets of immunofluorescence images were selected and are shown above.

## 3. Results

### 3.1. Morphological Assessment

**Figure 1:**
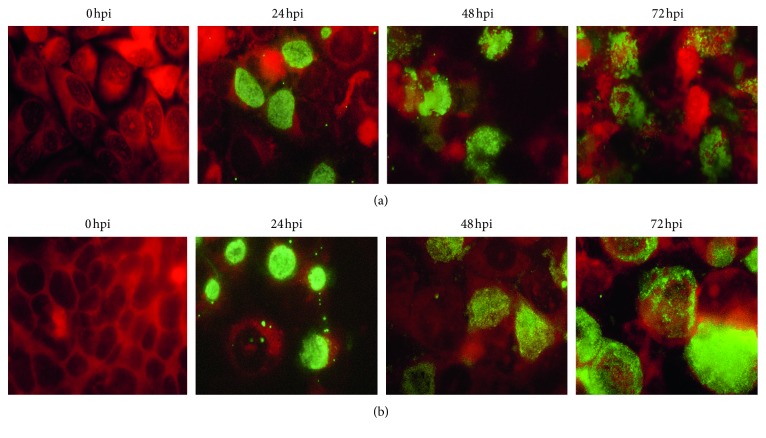
Immunofluoresence analysis of *C. trachomatis* propagation in (a) McCoy versus (b) CWR-R1 cells. McCoy and CWR-R1 cells were plated, grown, and harvested at “0”, 24, 48, and 78 hours after inoculation of *C. trachomatis*. The cells were stained with MOMP-specific antibody and photographed as described in Material and Methods.

The morphological characteristics of *C. trachomatis* growth in McCoy and CWR-R1 cells were monitored in immunofluorescence experiments conducted side by side. There was no difference in the attachment/entry of EB into McCoy and CWR-R1 cells at the early time points of infection corresponding to 6, 9, and 12 hpi (results not shown). Almost equal numbers of infected cells were seen in both cells lines at the different MOI used (0.5 and 1.0). Also, there was no statistical difference in the number of infected cells in the cell lines investigated. For example, at 24 hpi, the number of infected cells seen in the McCoy line at MOI = 1 was 26.3 (95% CI : 29.4/22.9), whereas the corresponding infectivity rate in CWR-R1 cells observed under the same conditions was 28.1 (95% CI : 30.1/21.4, *P* > 0.05). Despite the similar number of infected cells assessed by IF at all time points, the inclusion bodies developing in McCoy cells at the initial stage of infection (24 hpi) tended to be less mature, with a patchy and more granular pattern of staining. Moreover, there was greater variability in the pattern of inclusion body staining in McCoy cells. In contrast, the majority of CWR-R1 cells at this time point had a less variable and stronger pattern of staining. They contained more mature inclusion particles with higher IF density and a more globular appearance, suggesting altogether a better synchronization of infection (Figure 1). At the late stage of the infectious cycle (72 hpi), the difference in the intensity of IF staining of the inclusion bodies was less obvious. However, the majority of McCoy cells were disrupted with visible exocytosis of EB, whereas EB in the CWR-R1 cells appeared to be more contained within cell remnants.

**Figure 2:**
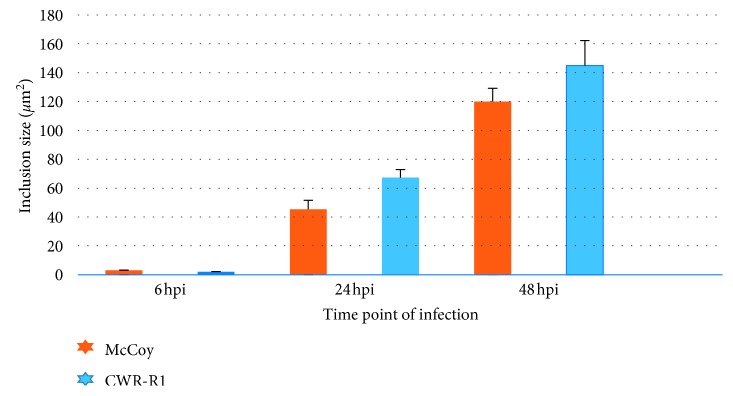
Inclusion body size in McCoy and CWR-R1 cells infected with *C. trachomatis*. McCoy and CWR-R1 cells were plated, grown, and harvested at 6, 24, 48, and 78 hours after inoculation of *C. trachomatis*. The cells were stained with MOMP-specific antibody, and inclusion size was measured as described in Material and Methods.

The results shown in Figure 2 document the difference in chlamydial inclusion size seen in both cell lines during the infectious cycle. As can be seen, under the culture conditions used, the inclusions in CWR-R1 cells were significantly larger (*P* < 0.05) than the inclusion bodies formed in McCoy cells at 24 and 48 hpi.

### 3.2. Infective Progeny Formation

**Figure 3:**
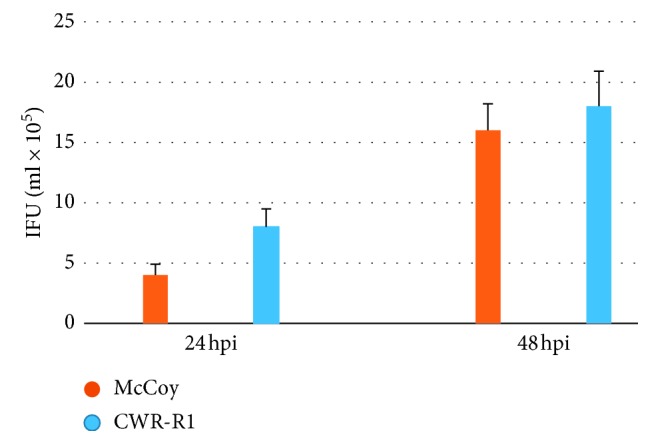
Infective progeny formation in McCoy and CWR-R1 cells infected with *C. trachomatis*. McCoy and CWR-R1 cells were plated, grown, and harvested at 24 and 48 hours after inoculation of *C. trachomatis*. Infective progeny formation was measured as described in Material and Methods in five different sets of the experiments, and average data with standard deviations are shown.

Figure 3 shows the difference in infective progeny formation in McCoy and CWR-R1 cells at 24 and 48 hpi. There was a statistically significant (*P* < 0.05) increase in the median reflecting infective progeny formation in CWR-R1 cells (7.7 × 10^5^, 95% CI : 9.1/6.3) in comparison with McCoy cells (4.6 × 10^5^, 95% CI : 5.4/3.7) at the early stage of infection (24 hpi). However, the later stage of chlamydial infection (48 hpi) was not accompanied by a statistically significant difference in infective progeny formation in the cell lines studied (*P* > 0.05).

### 3.3. *EUO* Expression

The results reported above reveal that the greatest differences in the infectious cycle of *C. trachomatis* in McCoy and CWR-R1 cells are evident at the initial stage of pathogen propagation in the host cells. Therefore, we decided to investigate next the mRNA levels for *EUO*, a key chlamydial gene controlling multiple gene transcription during the early stage of *C. trachomatis* propagation in eukaryotic cells [16].

**Figure 4:**
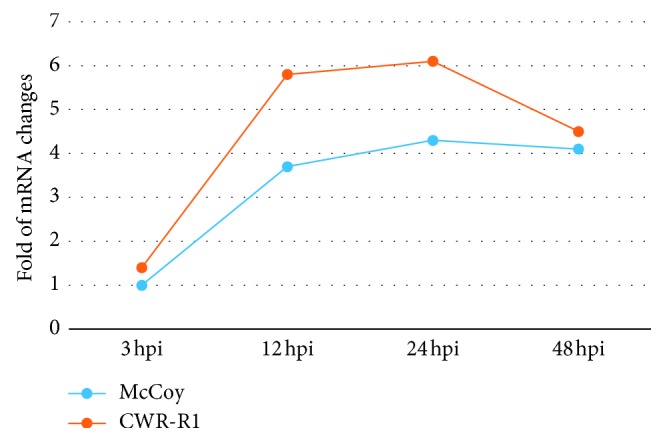
Folds of mRNA changes for *EUO* gene in McCoy and CWR-R1 cells infected with *C. trachomatis*. McCoy and CWR-R1 cells were plated, grown, and harvested at 3, 12, 24, and 48 hours after inoculation of *C. trachomatis*. EUO mRNA was measured as described in Material and Methods. The study was conducted 3 times. Most representative set of results is shown above.

As can be seen from Figure 4, the highest level of EUO expression in both cell lines was seen in our experiments at 12 hpi, and especially at 24 hpi. At both time points, *EUO* expression was noticeably higher in CWR-R1 cells than that in McCoy cells (by 2.1- and 1.8-folds, respectively). Interestingly, even at the earliest time point investigated (3 hpi), CWR-R1 cells had a higher level of copies for *EUO* mRNA (by 30%) than that in with McCoy cells. *EUO* mRNA remained almost equally elevated but subsided somewhat from the values measured at 24 hpi (up to 4.1- and 4.5-folds in McCoy and CWR-R1 cells, respectively) at 48 hpi.

### 3.4. Host Cell Cytokine mRNA Expression

Since *C. trachomatis* is known to affect cytokine gene expression in host cells during the postinfection period [17], we decided to investigate next the mRNA levels of IL-6 and FGF-2, two crucial proinflammatory cytokines, following exposure of McCoy and CWR-R1 cells to the pathogen.

**Figure 5:**
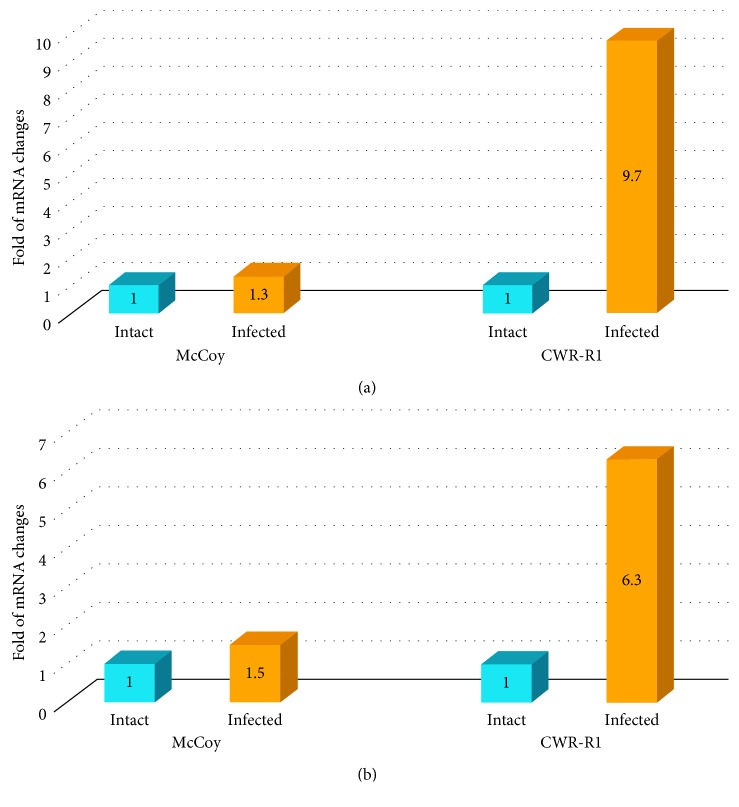
mRNA values for interleukin-6 (a) and fibroblast growth factor-2 (b) in McCoy and CWR-R1 cells infected with *C. trachomatis* AT 24 hpi. McCoy and CWR-R1 cells were plated, grown, and harvested 48 hours after inoculation of *C. trachomatis*. mRNAs for IL-6 and FGF-2 were measured as described in Material and Methods. The study was conducted 3 times. Most representative set of results is shown above.

As can be seen from Figure 5, both genes (IL-6 and FGF-2) are constitutively expressed in uninfected McCoy and CWR-R1 cells. Both proinflammatory mRNAs were upregulated in infected McCoy cells (up to 1.3- and 1.5-folds for IL-6 and FGF-2, respectively). However, increases were much more pronounced in the infected CWR-R1 cells. The mRNA level for IL-6 went up to 9.7-fold and mRNA for FGF-2 was upregulated by 6.3-fold as compared with mRNA values detected in the uninfected cells. Since the two cell lines used in the studies are of different origin (mouse versus human) and require different primer sets for mRNA quantification with possible differences in annealing efficiency, it would be questionable to conduct direct quantitative assessment of the mRNA responses in McCoy and CWR-R1 cells in terms other than fold increase over basal values detected for each cell line.

## 4. Discussion

Prostate cancer (PC) is the most common noncutaneous cancer in men and the third most common cause of cancer-related death in males residing in the USA and the UK [18]. Newly diagnosed individuals with localized PC have a high 10-year survival rate and a high chance of complete recovery whereas men with metastatic “aggressive” forms of PC tend to have much poorer outcomes [19, 20]. The molecular and cellular mechanisms of initiation, progression, and outcomes of PC are poorly understood. Chronic prostate inflammation (prostatitis), sedentary lifestyle, obesity, high-fat diet, and smoking are among epidemiological factors predisposing to PC [21, 22]. *Chlamydia trachomatis* is currently considered to be among common and frequent uropathogens causing chronic and difficult to treat bacterial prostatitis [23]. Although the frequency of confirmed cases of chlamydial prostatitis in the general population seems to be low, *C. trachomatis* can ascend the male urogenital tract and affect the bladder, prostate, seminal vesicles, epididymis, and testicles [24, 25].

In the present paper, we report that *C. trachomatis* can efficiently propagate in CWR-R1 cells, a PC cell line. This observation is in good agreement with results describing *C. trachomatis* growth in PC-3 cells, another PC cell line (26). Overall, the infectious cycle of chlamydial pathogen in CWR-R1 cells resembles the progression of *C. trachomatis* infection in McCoy cells, a classic cell line for chlamydial research, with a few important differences. First of all, the initial stage of *C. trachomatis* propagation in CWR-R1 cells (24 hpi) was characterized by larger inclusion bodies and higher intensity of IF staining of the infected cells as compared with McCoy cells. Moreover, there was a corresponding increase in infective progeny formation in CWR-R1 cells along with mRNA for EUO, a crucial gene controlling the early phase of the chlamydial development cycle. It remains an open question now if other developmental chlamydial genes (*incA* and *omcB*) studied in our previous work [15] have a different pattern of expression in McCoy and CWR-R1 cells during the later stages of infectious cycle. Interestingly, the changes in *EUO* expression between two cell lines studied were minimized and became statically insignificant at a later time point in the infectious cycle (48 hpi). Altogether, these data suggest that the early phase of *C. trachomatis* infection in CWR-R1 cells is accompanied by more efficient propagation of the pathogen as compared with the growth of *C. trachomatis* in McCoy cells. This conclusion comprises a novelty in our research. The underlying mechanism of these phenomena may be related to biochemical characteristics of PC cells and the metabolic requirements for *C. trachomatis* growth in the early stages of infection. CWR-R1 cells are known constitutively to express functional elements of mitogen-activated protein kinase, phosphatidylinositol-3 kinase, and Akt pathways and to display hyperactivated Akt which develops due to an Akt-dependent increase in oxidative phosphorylation [26, 27]. These changes are known to cause an intracellular excess of reactive oxygen species (ROS) which are an essential stimulus for initiation and progression of the developmental cycle of *C. trachomatis* in the urogenital system [3, 28]. It has been shown recently that high ROS production promotes chlamydial growth and infective progeny formation via activation of caspase-1 and hypoxia-inducible factor 1*α* [29]. Moreover, PC cell lines have been shown to have an impaired mitochondrial respiration rate and exemplify the Warburg effect [27, 30], resulting in enhanced glycolysis, lactogenesis, and inefficient ATP synthesis. However, as recently reported, moderate hypoxia is highly beneficial for the chlamydial growth cycle in cultured cells and promotes the infection rate of both *C. trachomatis and C. pneumoniae* [28]. Additionally, as shown lately [11] *C. trachomatis* can sustain its own energy needs during the infectious cycle at least in part via sodium-dependent synthesis of ATP. Therefore, high competition for ATP metabolic pathways in PC cells need not be a limiting factor for chlamydial cycle progression in PC cell lines and seems rather to be a factor activating chlamydial growth.

Another aspect of our results reveals that propagation of *C. trachomatis* in CWR-R1 cells leads to enhanced transcription of IL-6 and FGF-2, genes encoding two important pro-inflammatory cytokines. IL-6 is a well-known inducer of chemoresistance, progression, and metastatic transformation of PC [31, 32]. On the other hand, FGF-2 is reported to promote vascularization of primary PC tumors and formation of metastatic lesions [33]. Therefore, the pattern of changes in cytokine mRNA profile seen in CWR-R1 cells infected with *C. trachomatis* clearly leads to predisposition to the advancement of PC. Similar results have been reported by Sellami H et al. who described enhanced transcription of VEGF mRNA in PC-3 cells, another PC cell line, infected with *C. trachomatis* [34]. However, it has to be emphasized that these results are of highly questionable relevance to clinical practice. Although a limited body of epidemiological evidence places past urogenital infections and sexually transmitted infections among the risk factors for PC, there is no proven epidemiological link between chlamydiosis and PC yet [35]. Additional epidemiological analysis is required to evaluate the possible interplay between *C. trachomatis* infection and PC outcomes and to determine the relevance of the observed changes to clinical practice. Nevertheless, the results reported above and the cell culture system proposed in our study could be a useful tool in experiments exploring the role of impaired host cell mitochondrial function in chlamydial growth as well as in understanding the general biology of chlamydial infection.

## Data Availability

The original results will be available by request from corresponding author and will be placed on the public server https://lycotec.com.
